# Profil épidémiologique des fibromes utérins dans la région de Sidi Bel Abbes, Algérie

**DOI:** 10.11604/pamj.2013.15.7.2690

**Published:** 2013-05-06

**Authors:** Nourelhouda Chalal, Abbassia Demmouche

**Affiliations:** 1 Département de biologie. Faculté des sciences. Université Djillali Liabes. Sidi Bel Abbes. Algérie

**Keywords:** Fibromes, étude rétrospective, particularités épidémiologiques, Sidi Bel Abbes, fibroids, retrospective study, epidemiological features, Sidi Bel Abbes

## Abstract

**Introduction:**

Les léiomyomes ou fibromyomes plus communément dénommés fibromes, sont les tumeurs les plus répandues du tractus génital féminin. Ils affectent 20 à 25% des femmes en activité génitale. Notre étude vise à élucider la réalité de ce type de pathologie dans la région de sidi bel Abbes, nord-ouest d'Algérie.

**Méthodes:**

A travers une étude rétrospective allant du 1^er^ janvier 2008 au 1 mai 2011 portant sur les patientes opérées pour fibrome au sein de la maternité de Sidi Bel Abbes, nous avons relevé les particularités épidémiologiques et cliniques de cette pathologie.

**Résultats:**

Au total 323 cas de fibromes ont été recensés. La tranche d'âge la plus touchée varie de 40 à 44 ans dans une fourchette d'âge comprise entre 20 et 74 ans. 37.83% des patientes étaient nullipares. Une prédominance des patientes dont l'âge de la ménarche est précoce, a été retenue (60.3%). 3% des femmes ont présenté un terrain familial prédisposant. La symptomatologie était dominée par les hémorragies génitales (35%). La majorité des patientes (51.70%) présentaient un utérus polymyomateux dont la localisation des fibromes était principalement corporéale (96%), sous séreuse (43%). Le traitement était conservateur dans 71.82% des cas.

**Conclusion:**

Sur la base des résultats obtenus, la mise au point d'un programme national de sensibilisation et de dépistage précoce, s'impose

## Introduction

Les léiomyomes ou fibromyomes, plus communément dénommés fibromes, sont les tumeurs les plus répandues du tractus génital féminin. Ils touchent 20 à 25% des femmes en âge de procréer et sont 3 à 9 fois plus fréquents chez les femmes noires que chez les femmes blanches [1]. Plus fréquents chez les femmes afro-américaines, ils font leur apparition généralement après l'âge de 30 ans [1, 2].

De par leur biologie moléculaire peu révélatrice, l'étiologie de ce type de pathologies, demeure encore ambiguë. Cependant, leur apparition et leur croissance sont influencées par de nombreux facteurs dont: ‘estrogènes, progestatifs, facteurs de croissance et d'angiogénèse, prédisposition génétique, nulliparité volontaire, obésité, la première ménarche précoce [3, 4].

Souvent asymptomatiques, les léiomyomes décelés au cours d'un examen gynécologique systématique ou d'une des techniques d'imagerie pelvienne, présentent une symptomatologie diversifiée dont: ménorragies, métrorragies, douleurs pelviennes, pesanteur pelvienne, perception d'une masse pelvienne, infertilité ou à la suite de complications douloureuses, mécaniques ou hémorragiques [5]. Bien que leur dégénérescence sarcomateuse reste insignifiante, les fibromes s'avèrent de nos jours un véritable problème de santé publique. Notre étude a pour objectif de déterminer le profil épidémiologique de cette pathologie dans la région de Sidi Bel Abbes.

## Méthodes

A travers une étude rétrospective allant du 1^er^ janvier 2008 au 1 mai 2011 portant sur les patientes opérées pour fibrome au sein de la maternité de Sidi Bel Abbes, nous avons relevé les particularités épidémiologiques et cliniques de cette pathologie.

Le diagnostic de cette pathologie a été retenu en préopératoire sur des signes caractéristiques échographiques. Cette étude s'est basée sur un certain nombre de paramètres retenus suite à la consultation des dossiers médicaux, à savoir: l'âge, le statut matrimonial, la parité, les antécédents gynéco- obstétriques, les antécédents familiaux, l'âge de la ménarche, le motif de consultation, le nombre de fibromes, la localisation et la taille de la tumeur et le type d'intervention. L'analyse des données a été faite à l'aide du logiciel Excel 2007.

## Résultats

**Figure 1:**
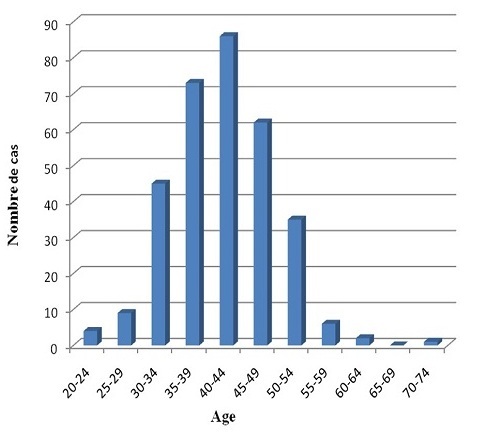
Répartition des patientes en fonction de l'âge

Durant cette période 323 cas ont été recensés, avec une prédominance des patientes dont l'âge est compris entre 40 et 44 ans, soit 86 cas. Par contre, Le nombre de cas diminue progressivement à la fois chez les femmes plus jeunes que chez les femmes plus âgées (Figure 1).

**Figure 2:**
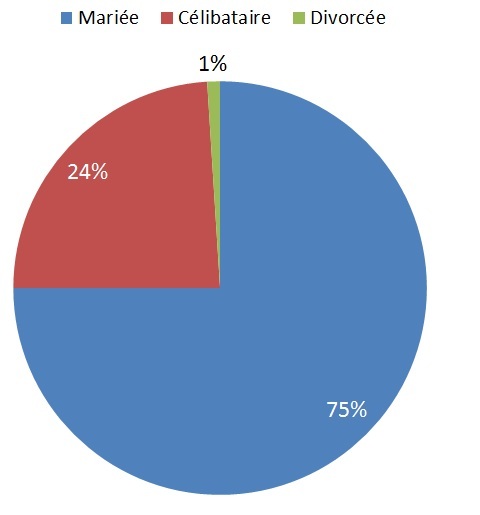
Répartition des patientes selon le statut matrimonial

Il est à noter que mis à part le pourcentage infime des femmes divorcées (1%), les trois quart des patientes sont des femmes mariées (75%). Les femmes célibataires ne représentent que 24%des patientes (Figure 2).

**Figure 3:**
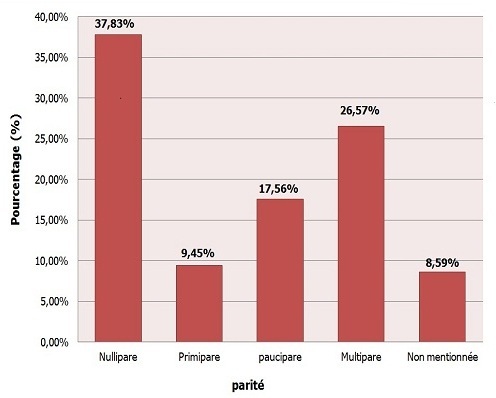
Répartition des patientes selon la parité

La répartition selon la parité, nous révèle une recrudescence de ce type de pathologies chez les femmes nullipares soit 37.83% par rapport aux femmes multipares soit 26.57%, (Figure 3). En dehors de ce critère, les avortements spontanés représentaient l'antécédent gynéco-obstétrique le plus fréquent dans 19.5% des cas (Tableau 1).

**Tableau 1:** Répartition des patientes selon les antécédents gynéco-obstétriques

Antécédents gynéco-obstétriques	Pourcentage (%)
Avortements spontanés	19.5%
Grossesses extra-utérines	1.54%
Curetage	6.81%
Césarienne	4.95%
Myomectomie	3.71%
kystectomie de l'ovaire	3.71%
Nodulectomie du sein	2.16%
Ligature des trompes	1%

**Figure 4:**
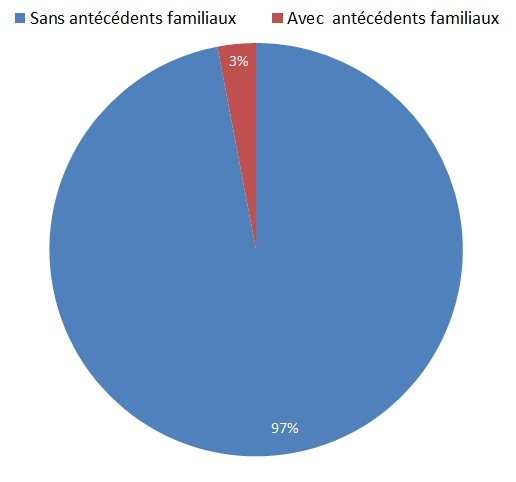
Répartition des patientes selon les antécédents familiaux

Seulement 3% des cas ont présenté un terrain familial prédisposant incluant: 3 patientes dont les mères avaient déjà des fibromes, 6 patientes dont les sœurs ont été touchées par cette pathologie, et on compte une seule patiente dont à la fois la mère et la sœur ont été atteintes (Figure 4).

**Figure 5:**
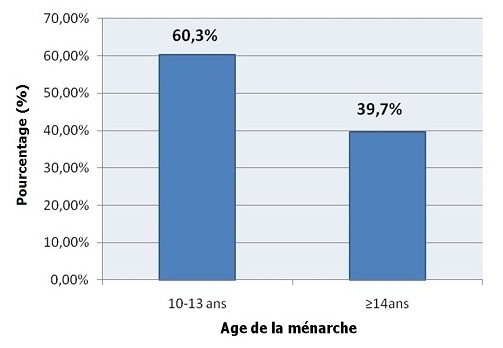
Répartition des patientes selon l'âge de la ménarche

La proportion des patientes dont l'âge de la ménarche est précoce (10 à 13 ans) est nettement plus élevée soit 60.3% que celle des autres dont l'âge de la ménarche est ≥14 ans soit 39.7% (Figure 5).

**Figure 6:**
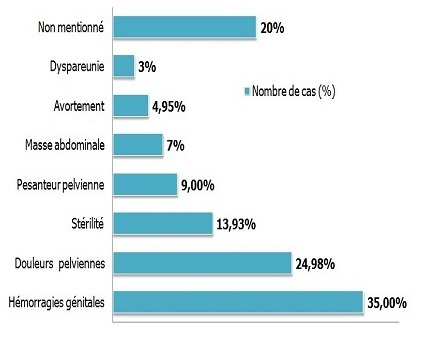
Répartition des patientes selon le motif de consultation

Les hémorragies génitales étaient le motif de consultation le plus fréquent soit 35% des cas (Figure 6).

La majorité des patientes (167cas soit 51.70%) avait un utérus polymyomateux. On compte 118 patientes soit 36.53% des cas qui n'avaient qu'un seul fibrome. Enfin 38 patientes soit 11.76% des cas n'en présentaient que deux (Tableau 2).

**Tableau 2:** Répartition selon le nombre, la localisation et la taille des fibromes

Paramètres	Pourcentage (%)
**Nombre de fibromes**	
1	36.53%
2	11.76%
≥3	51.70%
**Localisation des fibromes**	
Par rapport aux différents segments de l'utérus	
Corps	96%
Isthme	3%
Col	1%
Par rapport à la paroi utérine	
Sous séreux	43%
Sous muqueux	34%
Intramural	23%
**Diamètre moyen (cm)**	
<6	70.99%
6 à 10	20.28%
>10	8.73%

On remarque à travers notre étude une prédominance des fibromes du corps utérin (96%) en comparaison avec les fibromes isthmiques (3%) et cervicaux (1%). Cependant, selon la localisation par rapport à la paroi utérine les fibromes sous séreux sont les plus répandus (43%), (Tableau 2).

En fonction de la taille, la plus part des fibromes (70.99%) avait un diamètre moyen < 6cm. Ceux dont le diamètre moyen varie entre 6 à10 cm représentaient 20.28% et 8.73% de diamètre > 10cm (Tableau 2).

**Figure 7:**
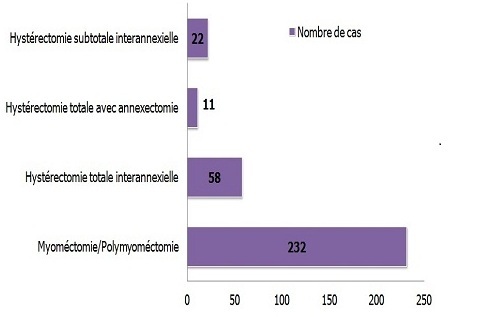
Répartition des patientes selon le type d'intervention

La majorité des patientes opérées (232 cas soit 71.82%) ont fait l'objet d'une myomectomie. 58 patientes soit 17.95% ont subi une hystérectomie totale interannexielle. D'autre part, on a opté pour une hystérectomie subtotale interannexielle pour 22 d'entre elles soit 6.81%. Cependant seulement 11 patientes soit 3.40% des cas ont été opérées par hystérectomie totale avec annexectomie (Figure 7).

## Discussion

L'étude qu'on a menée nous révèle que cette pathologie est fréquente dans la région de Sidi Bel Abbes. Elle apparait généralement après l'âge de 30 ans et régresse après la ménopause, du fait de la diminution du taux d'estrogènes [6, 7].

Les femmes nullipares sont plus exposées à cette pathologie par rapport aux femmes multipares ce qui appuie selon de nombreuses études, l'association parité- fibrome qui fait ressortir le caractère protecteur de la multiparité contre l'apparition des myomes utérins [6, 8–11].

D'autre part, d'après Vikhlyaeva (1995) qui a travaillé sur un échantillon de 97 patientes et 118 membres de leurs familles respectives, il existe une prédisposition familiale aux fibromes qui est 2.2 fois plus fréquente lorsque dans la famille au 1^er^ degré on trouve des femmes avec 2 fibromes ou plus [12].

Selon notre étude, l'âge précoce de la ménarche constitue un autre facteur de risque lié à l'apparition des fibromes. C'est ce qui concorde avec plusieurs études dont: Lumbiganon et al [6], Faerstein et al [13], Romieu et al [14], samadi et al [15], Sato et al [10], Rein et al [16]. L'impact des léiomyomes sur la fertilité demeure toujours controversé. En effet aucune étude rapportant le taux de grossesse comparatif en présence et en l'absence de myome, n'a été publiée.

La présence d'un myome a fortiori sous-muqueux ou interstitiel déformant la cavité utérine, avait un effet délétère sur les résultats du replacement embryonnaire. Le rôle des myomes utérins comme facteur causal d'infertilité, peut être expliqué par des mécanismes physiopathologiques, ainsi une obstruction des ostias tubaires, une distorsion importante de la cavité utérine obligeant un trajet plus long aux spermatozoïdes, empêchent la fécondation, une dystrophie endométriale due à un trouble de la vascularisation, à un déséquilibre œstroprogestatif, n'est pas favorable à une nidation [17].

De par leur cartographie et les complications gynécologiques qui en découlent, il serait souhaitable que les femmes en activité génitale et notamment celles présentant des antécédents gynéco-obstétriques ou un terrain familial prédisposant, fassent l'objet d'un suivi gynécologique périodique et régulier.

La prise en charge thérapeutique doit correspondre aux exigences de chaque patiente et viser à en soulager les symptômes. Néanmoins, tel que nous l'avons constaté, la myomectomie a été envisagée comme thérapie conservatrice de première intention, privilégiée par les patientes désireuses de préserver leur fertilité.

## Conclusion

Au terme de notre étude, nous relevons que cette pathologie est fréquente dans la région de sidi bel Abbes et s'avère un véritable problème de santé publique qu'il est indispensable de cerner par l'élaboration d'un programme national de sensibilisation et de dépistage précoce.
